# Cellular analysis of SOD1 protein-aggregation propensity and toxicity: a case of ALS with slow progression harboring homozygous *SOD1-D92G* mutation

**DOI:** 10.1038/s41598-022-16871-3

**Published:** 2022-07-25

**Authors:** Masanori Sawamura, Keiko Imamura, Rie Hikawa, Takako Enami, Ayako Nagahashi, Hodaka Yamakado, Hidenori Ichijo, Takao Fujisawa, Hirofumi Yamashita, Sumio Minamiyama, Misako Kaido, Hiromi Wada, Makoto Urushitani, Haruhisa Inoue, Naohiro Egawa, Ryosuke Takahashi

**Affiliations:** 1 Department of Neurology, Kyoto University Graduate School of Medicine, 54 Kawahara-cho, Shogoin, Sakyo-ku, Kyoto, 606-8507 Japan; 2 Center for iPS Cell Research and Application (CiRA), Kyoto University, Kyoto, Japan; 3 iPSC-Based Drug-Discovery Cellular Basis Development Team, RIKEN BioResource Research Center (BRC), Kyoto, Japan; 4 Medical-Risk Avoidance Based on iPS Cells Team, RIKEN Center for Advanced Intelligence Project (AIP), Kyoto, Japan; 5 Laboratory of Cell Signaling, Graduate School of Pharmaceutical Sciences, The University of Tokyo, Tokyo, Japan; 6 Department of Neurology, Japanese Red Cross Wakayama Medical Center, Wakayama, Japan; 7 Department of Neurology, Shiga University of Medical Science, Shiga, Japan; 8 Department of Neurology, Sakai City Medical Center, Osaka, Japan; 9 Karasuma Wada Clinic, Kyoto, Japan

**Keywords:** Diseases of the nervous system, Motor neuron disease, Neurodegeneration, Mechanisms of disease

## Abstract

Mutations within *Superoxide dismutase 1* (*SOD1*) cause amyotrophic lateral sclerosis (ALS), accounting for approximately 20% of familial cases. The pathological feature is a loss of motor neurons with enhanced formation of intracellular misfolded SOD1. Homozygous *SOD1-D90A* in familial ALS has been reported to show slow disease progression. Here, we reported a rare case of a slowly progressive ALS patient harboring a novel *SOD1* homozygous mutation *D92G* (*homD92G*). The neuronal cell line overexpressing SOD1-D92G showed a lower ratio of the insoluble/soluble fraction of SOD1 with fine aggregates of the misfolded SOD1 and lower cellular toxicity than those overexpressing SOD1-G93A, a mutation that generally causes rapid disease progression. Next, we analyzed spinal motor neurons derived from induced pluripotent stem cells (iPSC) of a healthy control subject and ALS patients carrying *SOD1-homD92G* or heterozygous *SOD1-L144FVX* mutation. Lower levels of misfolded SOD1 and cell loss were observed in the motor neurons differentiated from patient-derived iPSCs carrying *SOD1-homD92G* than in those carrying *SOD1-L144FVX*. Taken together, SOD1-homD92G has a lower propensity to aggregate and induce cellular toxicity than SOD1-G93A or SOD1-L144FVX, and these cellular phenotypes could be associated with the clinical course of slowly progressive ALS.

## Introduction

Amyotrophic lateral sclerosis (ALS) is a motor neuron disease characterized by selective neuronal death of both upper and lower motor neurons. ALS develops mainly in middle age or later, and the disease progresses relatively quickly and is usually fatal within 2–5 years^1^. Though the pathological mechanism of sporadic ALS is still unknown, 5–10% of cases are familial ALS (FALS)^1^.

Mutations of SOD1 are linked to approximately 20% of FALS cases^2,3^. The Japanese consortium for ALS research (JaCALS) reported that the frequencies of *SOD1*, *FUS/TLS*, *TARDBP*, and *VCP* variants in Japanese FALS were 35.9%, 7.7%, 2.6%, and 2.6%, respectively (total of 39 FALS patients)^4^. Another Japanese group reported that the frequencies of *SOD1, FUS/TLS, SETX, TARDBP, ANG,* and *OPTN* variants in FALS were 32%, 11%, 2%, 2%, 1%, and 1%, respectively (total of 111 FALS patients)^5^. In Japanese FALS, SOD1 variants are the most common mutation. A hexanucleotide repeat expansion in *C9orf72* was not found in these cohorts, whereas it was the most common mutation in Caucasian ALS patients^6–8^.

So far, more than 200 mutations of SOD1 have been described, and most of them are heterozygous mutations, the pathological mechanism of which is probably related to gain of a toxic function rather than loss of function. Previous studies have pointed out that part of the toxicity of mutant SOD1 may be involved in its aggregation. FALS patients carrying heterozygous *G93A* mutation for *SOD1* (*SOD1-G93A*) exhibit rapid progression of motor symptoms and die of pneumonia or respiratory insufficiency approximately 3 years after onset of symptoms^9^. In vitro SOD1-G93A protein has been reported to have a higher propensity to aggregate than wild-type SOD1 (SOD1-WT), leading to formation of cytoplasmic inclusions and cellular toxicity^10,11^. A FALS patient carrying *SOD1-L144FVX* has been reported with severe motor symptoms, resulting in death only 10 months after onset^12^. We have demonstrated that induced pluripotent stem cells (iPSC) of the patient carrying *SOD1-L144FVX* showed misfolded SOD1^13^.

A rare case of ALS showing slow progression that harbored a novel *SOD1* homozygous *D92G* mutation (*homD92G*) is presented. Protein-aggregation propensity and cellular toxicity were examined using cell lines and motor neurons derived from a patient carrying *SOD1-homD92G*.

## Results

### Identification of a novel *SOD1* homozygous mutation of *D92G* in an ALS patient with slow progression

A 54-year old woman noticed difficulty standing up from a chair. She felt difficulty grabbing objects with her right hand at the age of 60 years, and then the same symptom gradually progressed to the left hand. Her temperature sensation was diminished at the age of 62 years. These symptoms gradually worsened, and she recently could not walk without a cane. She visited our hospital at 71 years of age.

General cognitive function was preserved. Neurological findings showed muscle atrophy of both thenar muscles and thighs with fasciculations. Manual muscle testing (MMT) showed proximal dominant muscle weakness (MMT 2–3/V). Gowers sign was present. Deep tendon reflexes were increased on both side limbs. Babinski’s sign was present on the right side. Temperature sensation was diminished in the lower limbs.

**Figure 1:**
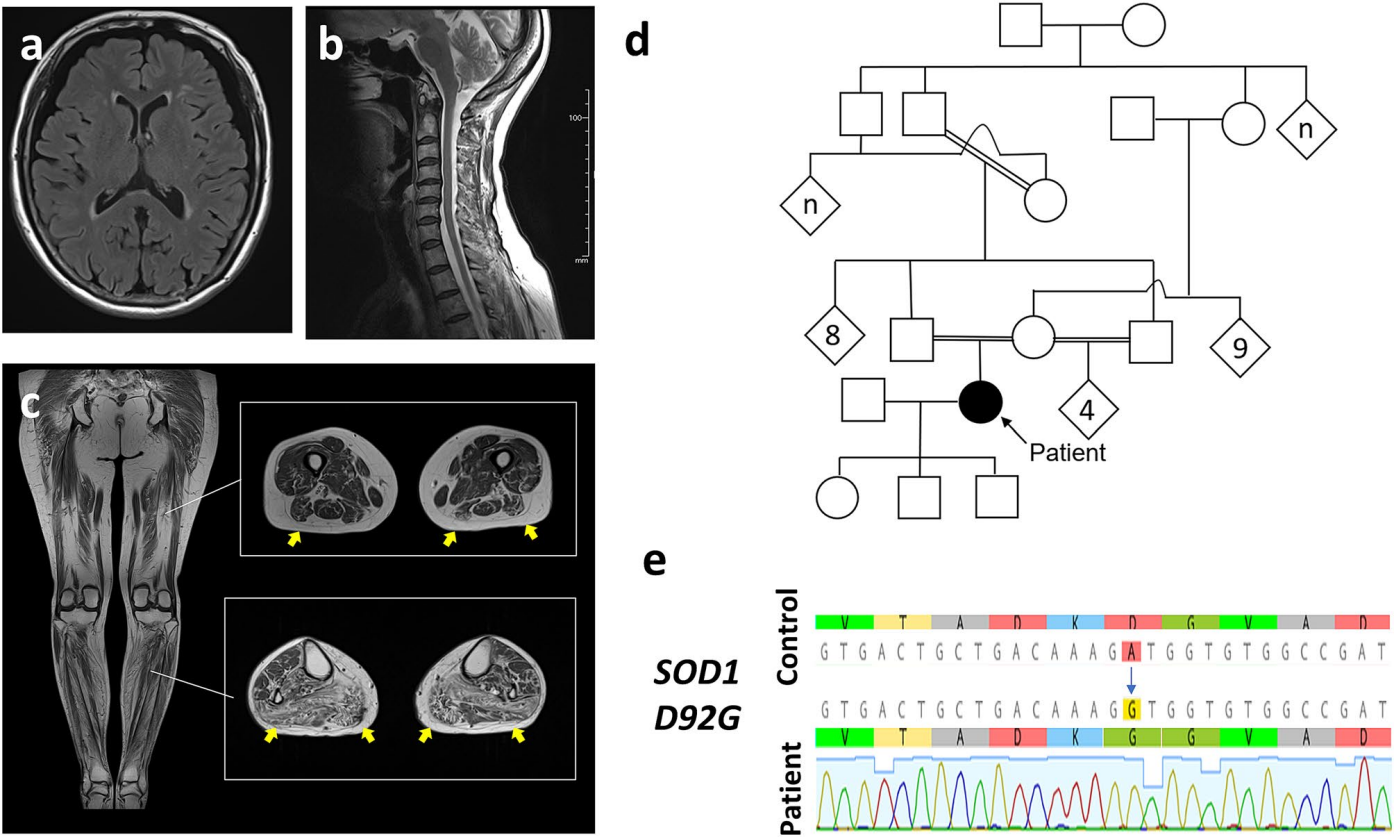
Clinical features of the ALS patient carrying a homozygous *D92G* mutation of *SOD1*. (**a**–**c**) Magnetic resonance imaging (MRI) of the brain, spinal cord, and lower limbs. There are no abnormalities on MRI of the brain (**a**) and spinal cord (**b**). MRI of the lower limbs shows apparent muscle atrophy and fatty change predominantly in the calves (**c**, yellow arrows). (**d**) The family history shows repeated consanguineous marriages, but there are no other patients in the family. (**e**) DNA sequencing shows homozygous *D92G* mutations of *SOD1*.

Magnetic resonance imaging (MRI) of the brain and spinal cord showed no specific abnormality (Fig. 1a,b), and MRI of the lower limbs demonstrated atrophy and high intensity on T1-weighted imaging (WI) in the calf (Fig. 1c). Electromyography showed acute and chronic neurogenic changes of the 1st dorsal interosseous, T7 paraspinal, tibialis anterior, and gastrocnemius muscles. The nerve conduction study showed decreased compound muscle action potentials (CMAPs) in the median and peroneal nerves and a mild decrease of the sensory nerve action potential (SNAP) in the sural nerve, but no evidence of conduction block or demyelination. Taken together, she was clinically diagnosed at the age of 71 years as having probable ALS according to the updated Awaji criteria^14,15^, though the progression of motor symptoms was slow compared to that of typical ALS patients. Her family history showed repeated consanguineous marriages, indicating autosomal recessive inheritance (Fig. 1d). DNA sequencing showed homozygous *D92G* mutation in *SOD1* gene (*homD92G*) (Fig. 1e). We performed whole exome sequencing using the patient’s sample to examine the responsible genes of FALS (Supplementary Table 1). The SOD1-D92G was the only mutation with clinical significance determined by ClinVar (https://www.ncbi.nlm.nih.gov/). Her parents had passed away without motor dysfunction. It is assumed that they could have been subclinical carriers with heterozygous SOD1-D92G mutation.

### In silico analysis of SOD1-D92G

To examine the structure-related protein propensity of SOD1-D92G, several in silico analyses were performed in comparisons with known FALS mutations, SOD1-L84F, N86S, D90A, G93A, and L126S. SOD1 mutations showing a rapid progression or severe phenotype, SOD1-L84F, N86S, G93A, and L126S showed predictive values of “probably damaging” or “deleterious” protein propensity on 3–4/4 software analyses (Table 1). On the other hand, SOD1 mutations showing a slow progression phenotype, SOD1-D90A and D92G, showed predictive values of “deleterious” protein propensity on 0–1/4 software analyses (Table 1). In addition, SOD1-D92G showed “deleterious” with a PROVEAN score of − 2.872 (cutoff = − 2.5), although the score was near the cutoff. Taken together, the severity of the phenotype in FALS with *SOD1* mutation may be correlated with the predicted value of protein propensity on in silico analysis.

**Table 1:** In silico analysis of FALS mutations.

Mutations	Inheritance	Onset	Phenotype	Duration	Prediction tool				References
					PANTHER	PROVEAN	SIFT	PloyPhen2	
L84F NP_000445: p. (Leu85Phe)	Homo	40	NA	Rapid progression 3 years	Deleterious (− 3.66439)	Deleterious (− 3.889)	Deleterious (0.05)	Probably damaging (1.0)	Boukaftane et al.^21^
N86S NP_000445: p. (Asn87Ser)	Homo	13	LMN	1 year	Deleterious (− 4.76358)	Deleterious (− 4.936)	Deleterious (0.0)	Probably damaging (1.0)	Hayward et al.^22^
D90A NP_000445: p. (Asp91Ala)	Homo	32–68	LMN	Slow progression	Neutral (− 2.31525)	Neutral (− 2.179)	Tolerated (0.16)	Benign (0.0)	Conforti et al.^29^
D92G NP_000445: p. (Asp93Gly)	Homo	54	UMN and LMN	Slow progression over 20 years	Neutral (− 2.33723)	Deleterious (− 2.6)	Tolerated (0.13)	Benign (0.0)	The present study
G93A NP_000445: p. (Gly94Ala)	Hetero	60.3 (55–63)	LMN	Rapid progression 3 years (2–4 years)	Neutral (− 2.95556)	Deleterious (− 5.447)	Deleterious (0.03)	Probably damaging (0.991)	Synofzik et al.^9^
L126S NP_000445: p. (Leu127Ser)	Homo	37	UMN and LMN	Rapid progression	Deleterious (− 5.6828)	Deleterious (− 5.672)	Deleterious (0.0)	Probably damaging (1.0)	Kato et al.^23^

### Low aggregation propensity and minor cell toxicity of the Neuro2a cell line overexpressing SOD1-D92G

**Figure 2:**
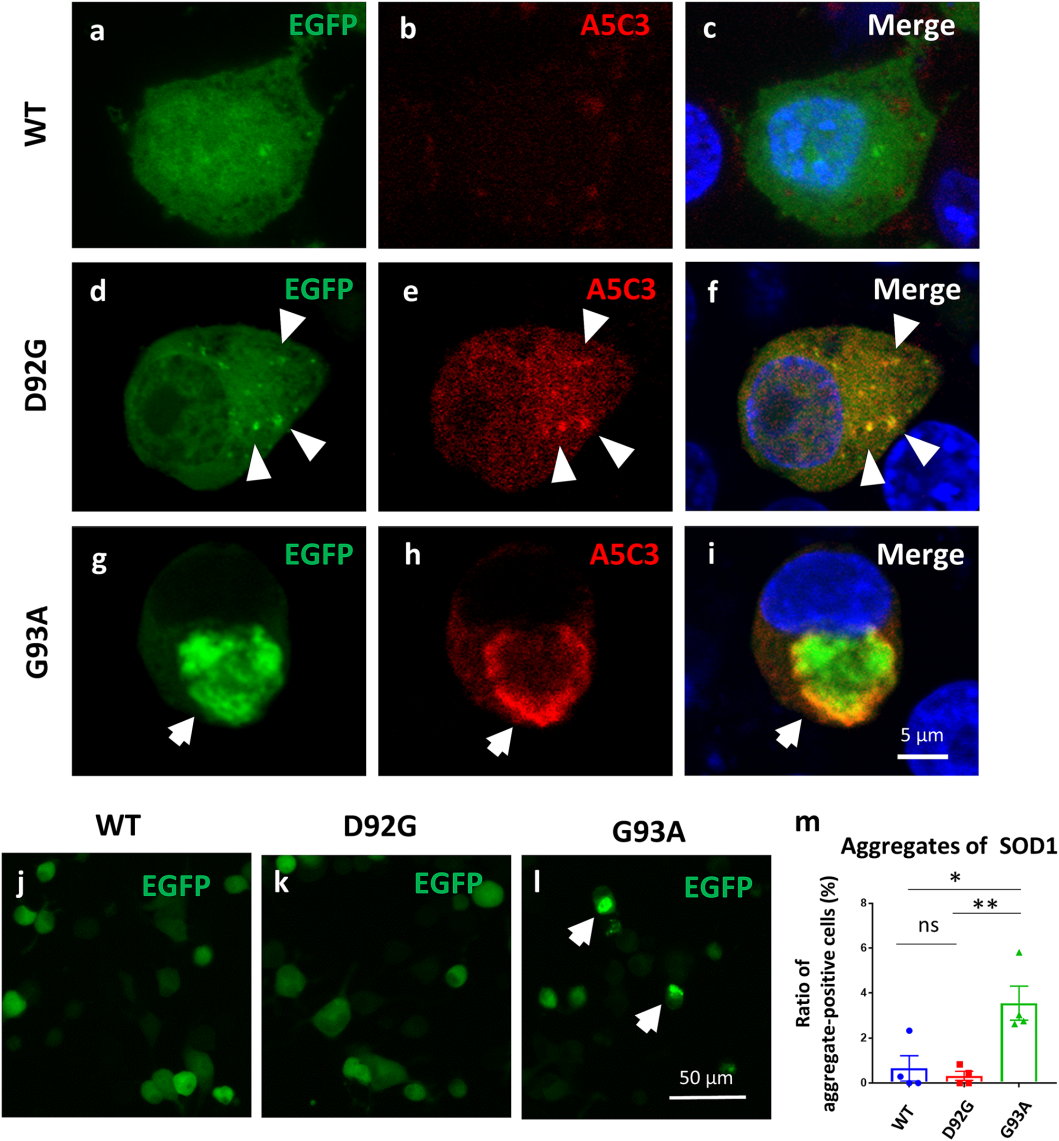
Analysis of aggregates of mutant SOD1. (**a**–**i**) Confocal microscopy images of SOD1-EGFP with immunostaining of misfolded SOD1. (**a**–**c**) SOD1-WT-EGFP (green) and misfolded SOD1 (A5C3, red). (**d**–**f**) SOD1-D92G-EGFP (green) and misfolded SOD1 (A5C3, red). Fine aggregates are observed in cells expressing SOD1-D92G-EGFP, which are immunostained with misfolded SOD1 antibody (arrow heads). (**g**–**i**) SOD1-G93A-EGFP (green) and misfolded SOD1 (A5C3, red). Round aggregates are observed in cells expressing SOD1-G93A-EGFP, which is immunostained with misfolded SOD1 antibody (arrows). Scale bar 5 µm. (**j**–**l**) There are no dense aggregates in cells expressing SOD1-WT/D92G-EGFP, whereas cells expressing SOD1-G93A-EGFP exhibit dense round aggregates. (**m**) Cells expressing SOD1-G93A-EGFP have significantly more dense round aggregates than cells expressing SOD1-WT/D92G-EGFP (p < 0.01) (n = 4). Scale bar 50 µm. Data are shown as means ± SD. One-way ANOVA (followed by Tukey’s test) is used for statistical analysis. *p < 0.05, **p < 0.01.

The aggregation property and cellular toxicity of SOD1-D92G were assessed in mouse neuroblastoma Neuro2a cells transfected with *pSOD1-D92G-EGFP* vector. As a positive control, *pSOD1-G93A-EGFP*, which mutation has shown aggregation phenotypes in in vitro cell lines, as well as in the motor neurons of patients and rodent models, was used^11,17^. No significant difference in transfection efficiencies was found among *pSOD1-WT-EGFP, D92G-EGFP,* and *G93A-EGFP* (Supplementary Fig. 1). Confocal microscopy showed large aggregates in cells expressing SOD1-G93A-EGFP (G93A), whereas fine aggregates were seen in cells expressing SOD1-D92G-EGFP (D92G) (Fig. 2d–i). Both aggregates were immunostained by monoclonal antibody to specifically detect misfolded SOD1 (A5C3)^18^. Cells expressing SOD1-WT-EGFP (WT) did not show misfolded aggregates (Fig. 2a–c). Low magnification images of G93A showed a dense round formation (EGFP-aggregate) in the cytoplasm (Fig. 2l), whereas most D92G and WT showed disperse formation (Fig. 2j,k). The ratio of the number of EGFP-aggregate positive cell relative to whole EGFP-expressing cells was significantly less in D92G than in G93A, whereas the aggregation propensity of D92G was similar to that of WT (Fig. 2m).

**Figure 3:**
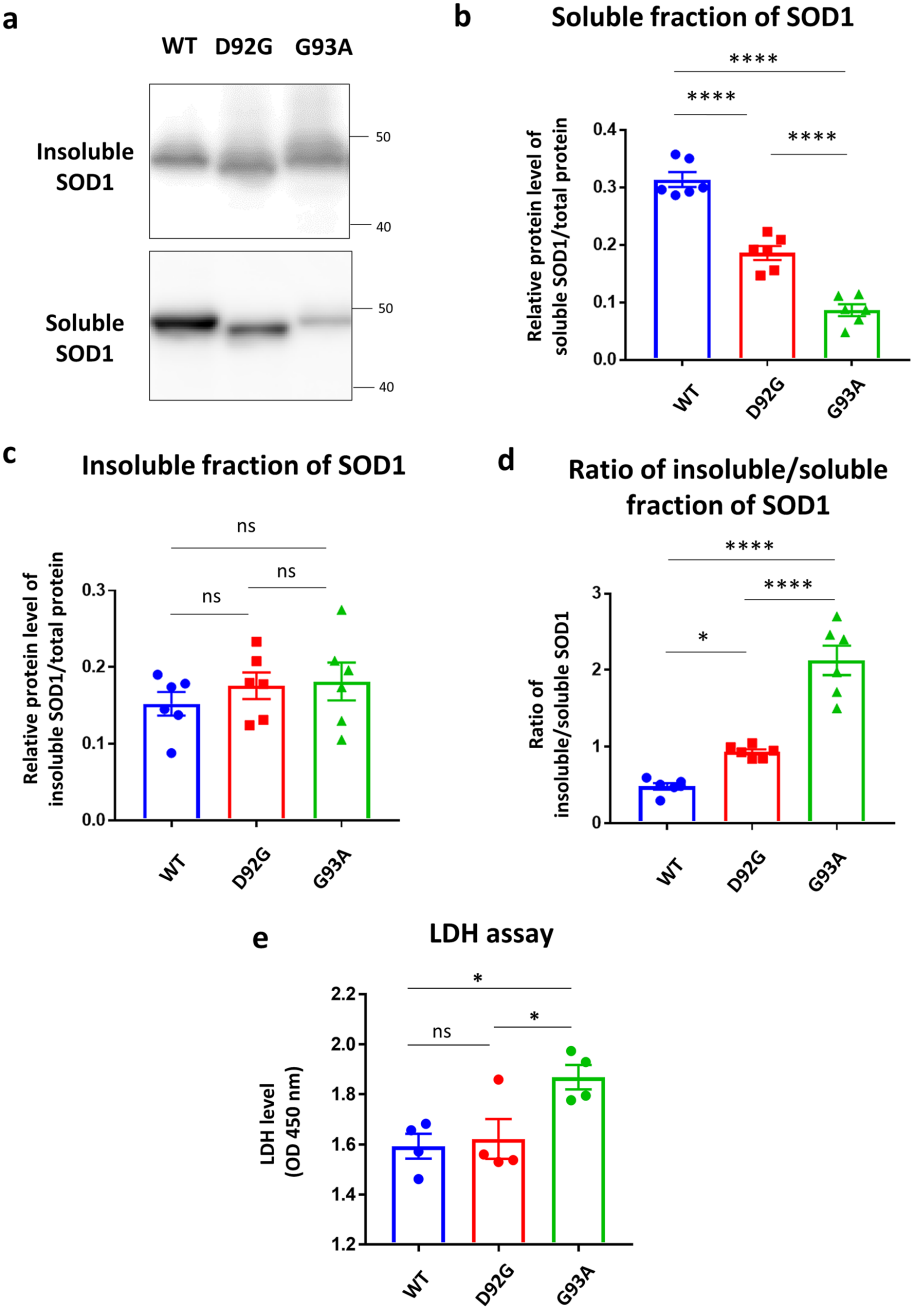
Western blotting analysis of mutant SOD1. (**a**) Representative western blotting images of SOD1 in soluble and insoluble fractions of cells expressing SOD1-WT, D92G, and G93A. Two panels were cropped from original blots presented by Supplementary Fig. 2. **(b**,**c**) Quantitative analysis of SOD1 in the soluble fraction (**b**) and insoluble fraction (**c**) of cells expressing SOD1-WT, D92G, and G93A (n = 6). (**d**) The ratio of insoluble/soluble fraction of SOD1 of cells expressing SOD1-WT, D92G, and G93A. (**e**) Cellular toxicity level in SOD1-WT, D92G, and G93A was determined by LDH (n = 4). Data are shown as means ± SD. One-way ANOVA (followed by Tukey’s test) was used for statistical analysis. *p < 0.05, ****p < 0.0001. *OD* optical density.

Next, immunoblotting analysis was performed. The soluble fraction of G93A was the most reduced among G93A, D92G, and WT, and the differences between them were significant (Fig. 3a,b). In contrast, the insoluble fraction of G93A was slightly increased among them (Fig. 3a,c). The relative ratio of the insoluble to the soluble fraction of G93A was the most increased. There were significant differences in the relative ratio between two groups among WT, D92G and G93A (Fig. 3d). The condition of D92G may be different from that of WT.

Finally, cellular toxicity was examined in each subtype. The LDH assay showed that the toxicity of G93A was significantly higher than of D92G and WT (p < 0.05), whereas there was no significant difference between D92G and WT (Fig. 3e). Therefore, D92G has a lower propensity to aggregate and lower toxicity than G93A.

### Minor misfolded SOD1 and neurodegenerative phenotype of the motor neurons derived from patient iPSCs carrying *SOD1-homD92G*

**Figure 4:**
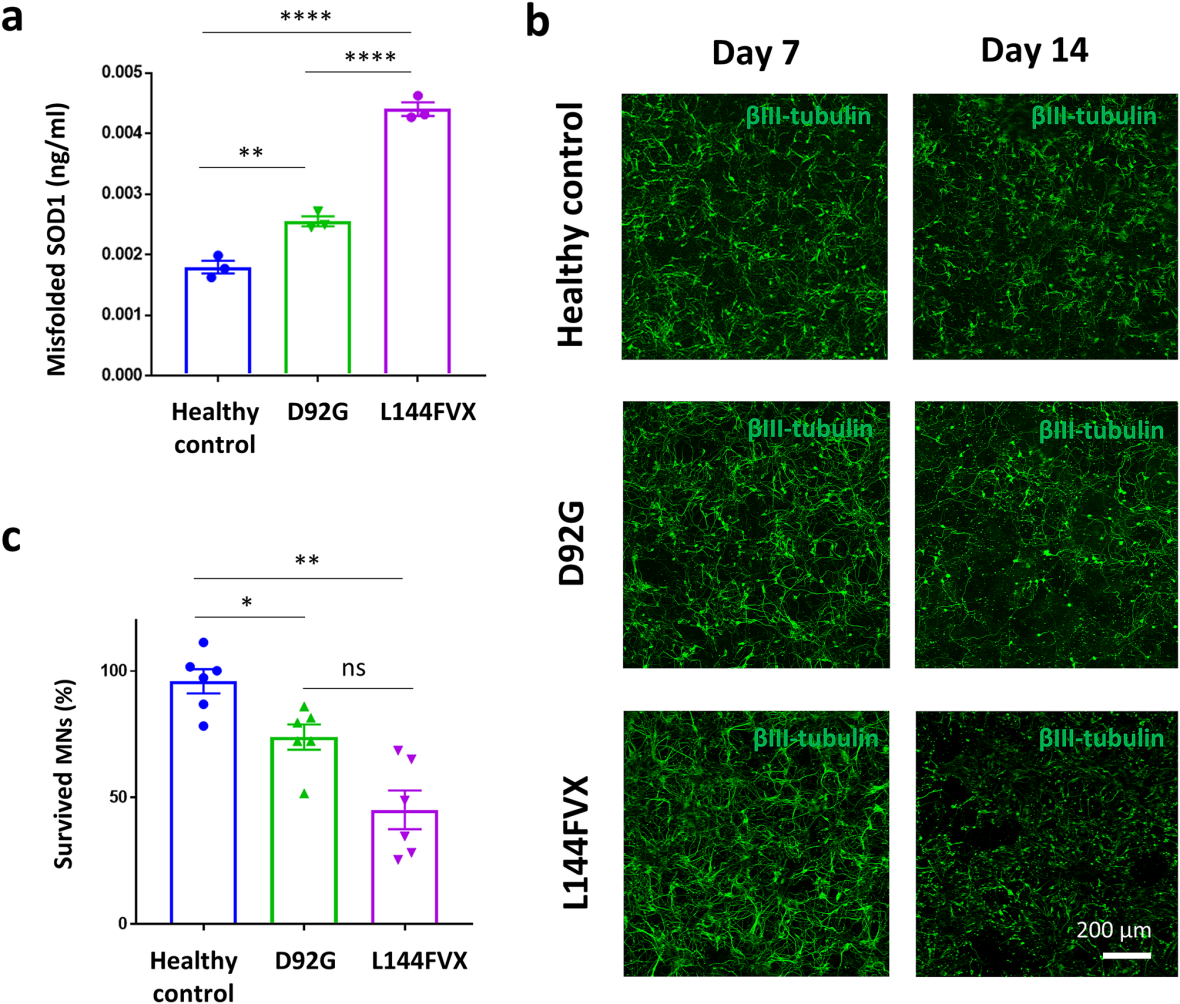
iPSC-derived motor neurons from patients with ALS. (**a**) Misfolded SOD1 in iPSC-derived motor neurons from a healthy control (201B7) and a patient with ALS carrying *SOD1-L144FVX* (A3316), and a patient with ALS carrying *SOD1-D92G* (A161EL1). (**b**) Representative images of iPSC-derived motor neurons derived from each patient on days 7 and 14. Scale bar 200 µm. (**c**) Evaluation of vulnerability of iPSC-derived motor neurons by measuring the number of neurons on days 7 and 14 (n = 6). One-way ANOVA was used for statistical analysis (followed by Tukey’s test). *p < 0.05, **p < 0.01, ****p < 0.0001.

Aggregation propensity and neurodegenerative phenotype were evaluated in an in vitro human cellular model using patient-derived motor neurons carrying *SOD1-homD92G*. We have established one iPSC line carrying D92G. We used one FALS-derived iPSC carrying heterozygous *SOD1-L144FVX*^13^ and healthy control-derived iPSCs 201B7^19^. The extent of misfolded SOD1 was examined using an ELISA assay detecting misfolded SOD1. The level of misfolded SOD1 was lower in motor neurons carrying *SOD1-homD92G* than in motor neurons carrying *SOD1-L144FVX* (Fig. 4a). Finally, the cellular vulnerability of patient-derived motor neurons was analyzed by a survival assay calculating cell viability expressed as a ratio of the number of neurons on Day 14 to the number of neurons on Day 7. The ratio of the number of survived motor neurons carrying *SOD1-homD92G* at Day 14 relative to those at Day 7 was significantly higher than that of *L144FVX* (Fig. 4b,c and Supplementary Table 2). Thus, motor neurons carrying *SOD1-homD92G* exhibited low aggregation propensity of misfolded SOD1 and a mild neurodegenerative phenotype compared to those carrying *L144FVX*.

## Discussion

It has been demonstrated that the clinical phenotype and prognosis of FALS harboring mutant *SOD1* were closely related to the mutated site of the gene^20^. Most *SOD1* mutations are heterozygous, but there have been reports that homozygous *SOD1* mutations showed juvenile-onset and rapid progression, such as a French case of FALS harboring *SOD1-L84F* mutation^21^, a juvenile-onset Pakistani case of FALS harboring *SOD1-N86S*^22^, and a Japanese case of FALS carrying *SOD1-L126S*^23^. Recent studies demonstrated that two ALS cases harboring homozygous *SOD1* truncating mutations (SOD1-C112Wfs*11) exhibited juvenile-onset and rapid progression accompanied by the loss of SOD1 activity^24,25^, suggesting that loss of *SOD1* function could be associated with the severe clinical course of ALS.

The present study is the first report of a case of FALS carrying *homSOD1-D92G* that showed onset in middle age and very slow progression. In a similar case, homozygous *SOD1-D90A* in FALS was reported to show slow progression in a Swedish/Finnish population^26^. The present in silico analyses showed that both SOD1-D90A and D92G had less “pathogenic” protein propensity than L84F, N86S, and L126S, of which homozygous mutations are linked with a severe clinical course. This suggests that protein structure-related properties in the various mutations of *SOD1* could explain the different propensities to aggregate and the clinical prognosis of *SOD1*-related FALS. Further studies are needed to elucidate the mechanism how these *SOD1* mutations could determine the clinical course of each form of ALS. To investigate the causal relationship between protein propensity from a mutation of *SOD1* gene and a neurodegenerative phenotype characterized by loss of motor neurons, protein aggregation, solubility and cellular vulnerability were examined using Neuro2a cells and patient-derived motor neurons. We found that SOD1-D92G is slightly more prone to aggregate than WT, but less prone to aggregate and less cytotoxic than G93A.

There are several limitations in this study. First, detailed pathological assessment using postmortem human samples of motor neurons was not performed. Further research is needed to determine whether mild motor neuron loss and aggregation could contribute to slower disease progression. A major barrier in the study of neurological disorders including ALS is that it is difficult to perform a highly invasive biopsy. It is more difficult to obtain the central nervous system (CNS) tissue, which is vulnerable to postmortem degeneration, suitable for biochemistry and molecular biology from autopsy. To overcome these barriers, we generated iPSC-derived motor neuron which has been reported to recapitulate the post-mortem CNS pathology in neurodegenerative disease^27^. Second, the mechanism of how homozygosity of *SOD1-D92G* could exert more toxicity than heterozygosity could not be elucidated. A deeper analysis (e.g. all-atom Molecular Dynamics simulation) of how the *homD92G* mutation affected the SOD1 dimer structure and how it could alter potential interactions with other proteins seems necessary to further comprehend the slow-progressing phenotype of this novel gene variant. Since there were no ALS cases in the presumed heterozygous parental generation, it was thought that the *SOD1-D92G* acquired ALS disease propensity only in homozygotes. A gene-correction study using patient-derived iPSCs might show the more precise cellular phenotypes of heterozygous and homozygous *SOD1-D92G*.

This study showed a relationship between the mutant SOD1 aggregation propensity and FALS prognosis in silico and a relationship between the SOD1 aggregation propensity and cell fragility in vitro. Future therapeutic approaches to regulate the aggregation propensity of SOD1 may alter the progression and prognosis of *SOD1*-related disease.

## Materials and methods

### Ethics statements and consent to participate

This study was approved by the Kyoto University Graduate School of Medicine Ethical Committee (G1178, R91). Written, informed consent was obtained from the participants prior to inclusion in the study. Samples from the participants were identified by numbers, not by names. All methods were performed in accordance with the relevant guidelines and regulations.

### MRI data acquisition

MRI scans were performed using the 3 T Magnetom Prisma or Avanto system for the brain or muscles, respectively (Siemens, Erlangen, Germany). Data analysis was performed with Centricity PACS 4.0 (GE Healthcare, Chicago, IL, USA).

### DNA sequencing

DNA was extracted from a blood sample, and previously reported primers were used^28^. The exons of *SOD1* were amplified and Sanger sequencing was performed on 3730xl DNA Analyzer (Thermo Fisher Scientific, Waltham, MA, USA) with Thermo Fisher ScientificTerminator v3.1 Cycle Sequencing Kit (Thermo Fisher Scientific). A novel *homD92G* mutation in the *SOD1* gene was identified (Fig. 1).

### Whole exome sequencing

For whole-exome sequencing, genomic DNA was extracted, and 1 μg was sheared and used for the construction of a paired-end sequencing library as described in the protocol provided by Illumina. Enrichment of exonic sequences was then performed for each library using the SureSelect Human All Exon V6 (Agilent Technologies Inc., Santa Clara, CA, USA) following the manufacturer’s instructions. Libraries for whole-exome sequencing were sequenced with a NovaSeq 6000 (Illumina Inc., San Diego, CA, USA).

### In silico analysis

The effects of the newly detected *SOD1-homD92G* and previously reported mutations of FALS cases (*SOD1-L84F, N86S, D90A, D92G, G93A, and L126S*) were analyzed with Mutation Taster (http://www.mutationtaster.org), Sorting Intolerant from Tolerant (SIFT, https://sift.bii.a-star.edu.sg/), PolyPhen-2 (http://genetics.bwh.harvard.edu/pph2/), PROVEAN (http://provean.jcvi.org/index.php), and PANTHER (http://www.pantherdb.org/).

### Plasmids and cell culture

KOD-Plus-Mutagenesis kits (TOYOBO, Osaka, Japan) were used to generate pSOD1-D92G-EGFP and pSOD1-G93A-EGFP plasmids. The mouse neuroblastoma Neuro2a cells were plated onto 24-well plastic plates (Greiner Bio-One, Kremsmünster, Austria) or glass-bottom dishes (Matsunami, Osaka, Japan), and maintained in Dulbecco’s modified Eagle’s medium (DMEM) (Wako, Tokyo, Japan) with 5% fetal bovine serum (FBS) (Thermo Fisher Scientific). The Neuro2a cells were transfected with plasmid carrying pSOD1-(WT/D92G/G93A)-EGFP using lipofectamine polyethyleneimine “MAX” PEI (Polysciences, Warrington, UK) reagent according to the manufacturer’s protocol. After overnight incubation, the medium was changed with new growth medium. At 48 h after transfection, the cells were fixed with 4% paraformaldehyde in phosphate-buffered saline (PBS). After 10-min incubation with PBS/0.1% Tween, the samples were incubated overnight at 4 °C with primary antibodies against misfolded SOD1 (A5C3, 1:200, MEDIMABS, Montreal, Canada). The samples were subsequently incubated with donkey-derived secondary antibodies (Alexa Fluor 594, 1:1000, Thermo Fisher Scientific) for 1 h at room temperature, and they were then covered with antifade mounting medium, VECTASHIELD^®^ with DAPI, (Vector Laboratories, Burlingame, CA, USA). High magnification images were acquired with an FV-1000 confocal laser scanning microscope (Olympus, Tokyo, Japan). SOD1-EGFP aggregates were observed with a BZ-X710 fluorescence microscope (KEYENCE, Osaka, Japan). A ‘SOD1-EGFP-aggregate’ was defined as a dense round formation more than 10 µm in diameter and counted in a double-blind manner. An ‘aggregate-positive cell’ was defined as a cell having an ‘EGFP-aggregate’. The ratio of the number of ‘aggregate-positive cells’ to that of whole EGFP-expressing cells was calculated.

### Western-blotting (WB)

The Neuro2a cells were plated onto 6-well plastic plates (Greiner Bio-One). The Neuro2a cells were transfected with the plasmid carrying *SOD1-*EGFP (*WT, D92G*, or *G93A*) using LTX (Thermo Fisher Scientific) reagent according to the manufacturer’s protocol. After overnight incubation, the medium was changed with new growth medium. At 48 h after transfection, the cells in 6-well dishes were washed twice with PBS and then scraped in 300 µl of PBS. The cells were sonicated (Cosmo Bio, Bioruptor, Tokyo, Japan) for 5 min and shaken for 15 min at 4 °C, and then the lysates were centrifuged at 12,000*g* for 10 min at 4 °C. The resulting pellet was resolved in 20 µl of 2% SDS as the PBS-insoluble fraction, and the supernatant was designated the PBS-soluble fraction. These lysates were incubated in 2 × SDS sample buffer and boiled at 95 °C for 5 min. One-fourth of the insoluble fraction and 1/60 of the soluble fraction were processed for the detection of SOD1. These proteins (PBS-soluble 5 µl, PBS-insoluble 5 µl) were loaded onto SuperSep™ Ace 10–20% (Wako, Tokyo, Japan). The proteins were then transferred onto polyvinylidene difluoride membranes and blocked with 5% skim milk in Tris-buffered saline Tween-20 (TBST) for 30 min. The membranes were incubated with an anti-SOD1 primary antibody (Enzo Life Science abi-sod-100F, 1:1000) at 4 °C overnight. Next, the membranes were incubated for 1 h at room temperature with a horseradish peroxidase secondary antibody (Santa Cruz #sc-2005, 1:10,000), and the protein bands were visualized using ECL Western Blotting Substrate (Thermo Fisher Scientific). Chemiluminescent signals were detected using an Amersham Imager 600 imager (GE Healthcare, Chicago, IL, USA). To evaluate the protein levels, these bands were analyzed with ImageJ ver. 1.50i (https://imagej.nih.gov/ij/). Total protein was measured by Coomassie Brilliant Blue staining (CBB Stain One, Nacalai Tesque, Kyoto, Japan). The insoluble fraction ratio was calculated as I/S, where S is the band intensity of the soluble fraction and I of the insoluble fraction.

### Cell viability assay

Neuro2a cells were plated onto 24-well plastic plates. Cell viability was evaluated with the LDH assay kit (Dojindo, Tokyo, Japan) 48 h after transfection. The LDH assay was performed according to the manufacturer’s protocol.

### Generation of human iPSCs

Human iPSCs were generated from peripheral blood mononuclear cells (PBMCs) using episomal vectors (*Sox2, Klf4, Oct3/4, L-Myc, Lin28*, and *p53-shRNA*) as reported previously^29^ and cultured by a feeder-free culture system with StemFit (Ajinomoto, Tokyo, Japan). Karyotype analysis of iPSCs was conducted by LSI Medience (Tokyo, Japan). Established iPSCs were cultured under feeder-free conditions on iMatrix (Nippi, Tokyo, Japan)-coated plates with StemFit AK01 (Ajinomoto).

### Generation of motor neurons

Motor neurons were generated from iPSCs as previously described^13^. Briefly, iPSCs carrying the tetracycline-inducible motor neuron differentiation cassette containing *Lhx3*, *Ngn2*, and *Isl1* (LNI cassette) under control of the tetracycline operator were established*.* The iPSCs were dissociated to single cells using Accumax and plated onto Matrigel-coated 96-well plates with the Neuronal Medium containing DMEM/F12 (Thermo Fisher Scientific), N2 (Thermo Fisher Scientific) containing 1 μM retinoic acid (Sigma), 1 μM Smoothened Agonist (SAG), 10 ng/ml BDNF (R&D Systems, Minneapolis, MN, USA), 10 ng/ml GDNF (R&D Systems), and 10 ng/ml NT-3 (R&D Systems) with 1 μg/ml doxycycline (TAKARA, Kusatsu, Japan), and cultured for 7 days.

### Motor neuron survival assay

The iPSCs derived motor neurons were cultured on iMatrix-coated 96-well plates (BD Bioscience, San Jose, CA) for 7 days and 14 days. The number of surviving motor neurons stained with the βIII-tubulin antibody was quantified by IN Cell Analyzer 6000 and IN Cell Developer toolbox software 1.9, and the ratio of surviving neurons on day 14 to those on day 7 was shown as a percent, as previously described^13^.

### Enzyme-linked immunosorbent assay (ELISA)

For ELISA of misfolded SOD1, motor neurons on day 7 were harvested and dissolved in buffer containing 1% Triton-X, 0.5% deoxycholate, 50 mM Tris–HCl, 1 mM EDTA, 0.1% SDS, 150 mM NaCl, 0.1% sodium deoxycholate, protease inhibitor (Roche), and phosphatase inhibitor (Roche). Samples were centrifuged at 13,000×*g* for 15 min at 4 °C. Then, 96-well plates (Thermo Fisher Scientific) were coated with 3 μg/ml MS785 antibody in 0.05 M sodium carbonate buffer at 4 °C overnight. After washing and blocking with TBS-T containing 1% BSA, 200 μg protein/100 µl of samples were added, and incubation was carried out for 2 h at room temperature. Recombinant mutant SOD1 protein (G93A) was used to obtain a standard curve. For detection, the plates were incubated with 3 μg/ml anti-SOD1 antibody (ENZO), followed by sheep anti-rabbit IgG F(ab)’2 fragment linked to horseradish peroxidase (1:3000; GE Healthcare). After incubation with tetramethylbenzidine solution (BD Bioscience) at room temperature for 30 min, absorbance at 450 nm was measured by VersaMax (Molecular Device, Sunnyvale, CA, USA).

## Supplementary Information

Supplementary Information.

## Data Availability

Web resources: SIFT (https://sift.bii.a-star.edu.sg/), PolyPhen-2 (http://genetics.bwh.harvard.edu/pph2/), PROVEAN (http://provean.jcvi.org/index.php), and PANTHER (http://www.pantherdb.org/). The datasets in the current study are available in the UniProtKB repository (https://www.uniprot.org/) (accession no. P00441).
