# Supplementary material for: Cellular analysis of SOD1 protein-aggregation propensity and toxicity: a case of ALS with slow progression harboring homozygous SOD1-D92G mutation
Source: Sci Rep. 2022 Jul 25;12:12636. doi: 10.1038/s41598-022-16871-3 (PMC9314329; doi:10.1038/s41598-022-16871-3)
Supplement: Supplementary file 1 — Supplementary Information. [file 41598_2022_16871_MOESM1_ESM.pdf]

Supplementary Figure 1

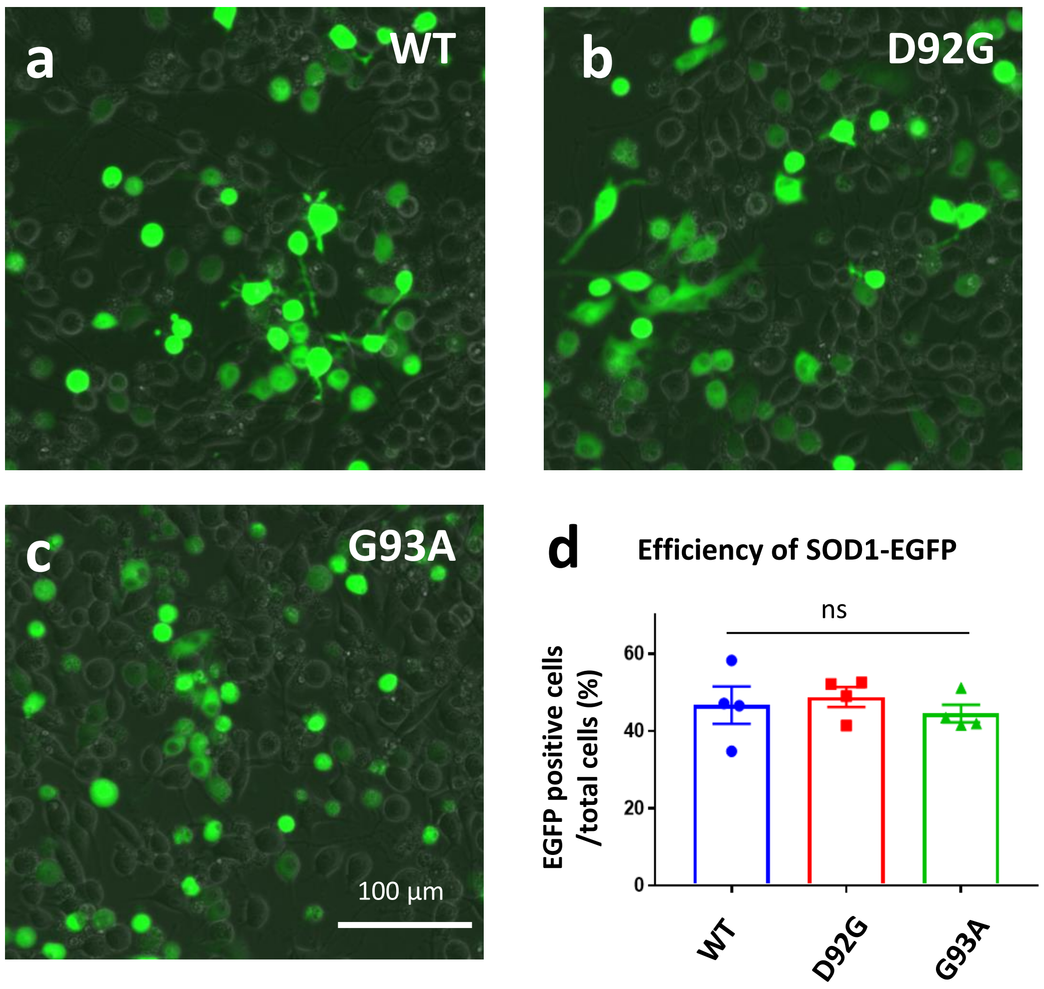

Supplementary Fig. 1. Transfection efficiency of *pSOD1-WT, D92G, and G93A-EGFP.*

(a–c) Representative images of Neuro2a cells transfected with *pSOD1-WT, D92G, and G93A-EGFP*.

(d) The ratio of EGFP positive cells per total cells (n=4). One-way ANOVA (followed by Tukey's test) is used for statistical analysis.

Supplementary Figure 2

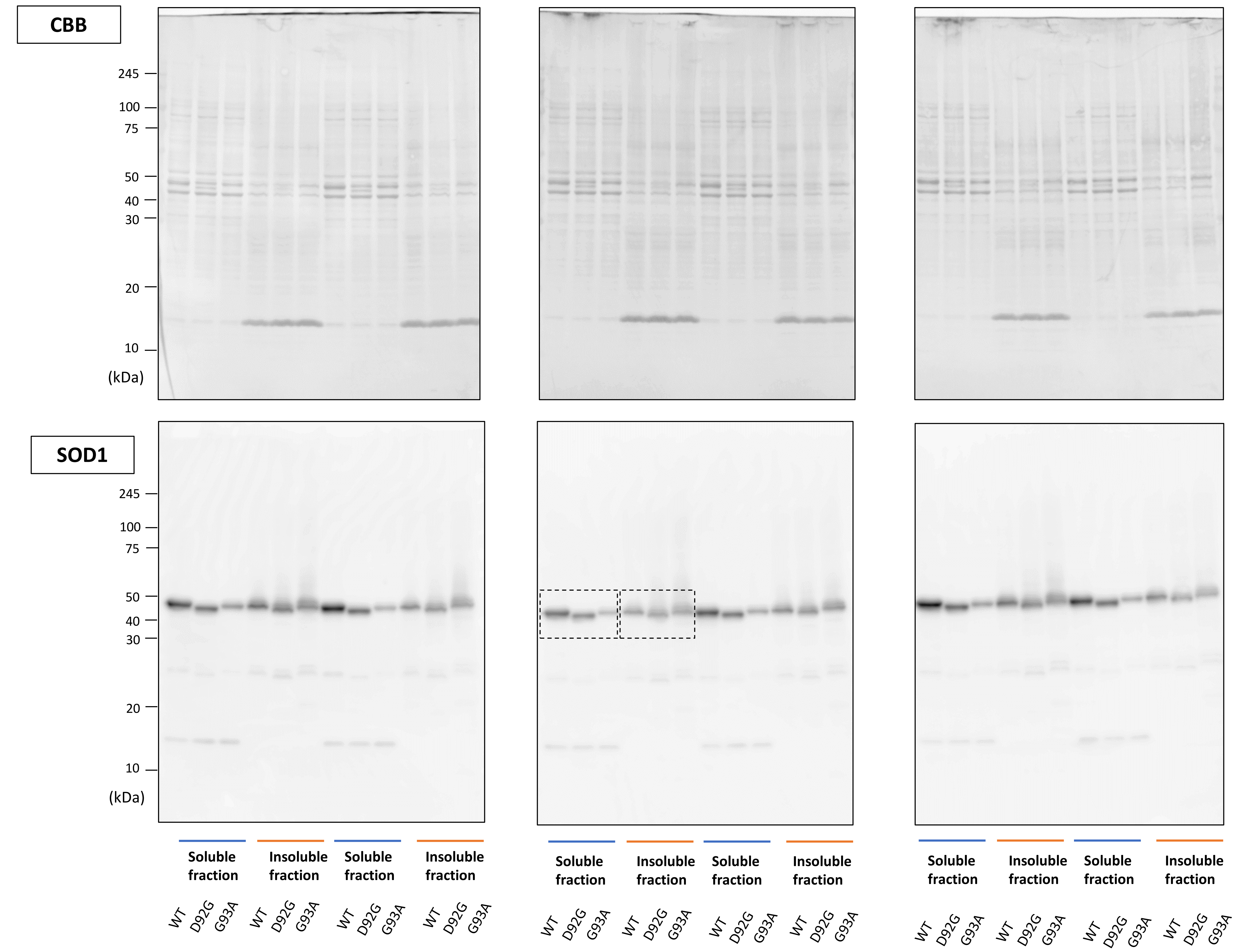

Supplementary Fig. 2. Whole original membranes in Western blot analysis

Whole original membranes in Western blot of SOD1 and Coomassie Brilliant Blue (CBB) staining. Representative western blot for SOD1 of soluble and insoluble fraction (dashed rectangles) equivalent to Fig. 3a.

Supplementary Table 1. The candidate genes of familial ALS.

Disease name	Gene name
ALS1	Cu/Zn–superoxide dismutase (SOD1)
ALS2	alsin
ALS4	senataxin (SETX)
ALS5	SPG11
ALS6	Fused in sarcoma/translocated in liposarcoma (FUS/TLS)
ALS8	Vesicle associated membrane protein-associated protein B (VAPB)
ALS9	angiogenin (ANG)
ALS10	transactive response -DNA binding protein (TARDBP)
ALS11	FIG4
ALS12	optineurin (OPTN)
ALS13	ataxin2 (ATXN2)
ALS14	Vasolin-containing protein (VCP)
ALS15	Ubiquillin 2 (UBQL2)
ALS16	Sigma non-opioid receptor 1 (SIGMAR1)
ALS17	Chromatin-modifying protein 2B (CHMP2B)
ALS18	Profilin1(PFN1)
ALS19	Erb-B2 Receptor Tyrosine Kinase 4 (ERBB4)
ALS20	Heterogenous nuclear ribonucleotide protein A1 (HNRNPA1)
ALS21	Matrin 3 (MATR3)
ALS22	Tubulin Alpha 4A (TUBa4A)
FTD-ALS1	C9orf72
FTD-ALS2	CHCHD10
FTD-ALS3	SQSTM1 (p62)
FTD-ALS4	TBK1

Supplementary Table 2. Number of surviving motor neurons.

	Number of neurons / well (mean ± SE)	
	Day 7	Day 14
Healthy control	1737.5 ± 66.8	1668.0 ± 82.9
SOD1-D92G	1374.7 ± 76.7	1016.7 ± 68.1
SOD1-L144FVX	1868.8 ± 172.6	844.8 ± 142.7
